# Sleep and light exposure across different levels of urbanisation in Brazilian communities

**DOI:** 10.1038/s41598-018-29494-4

**Published:** 2018-07-30

**Authors:** Luísa K. Pilz, Rosa Levandovski, Melissa A. B. Oliveira, Maria Paz Hidalgo, Till Roenneberg

**Affiliations:** 1 Laboratório de Cronobiologia e Sono, Hospital de Clínicas de Porto Alegre (HCPA)/Universidade Federal do Rio Grande do Sul (UFRGS), Porto Alegre, RS Brazil; 2 Programa de Pós-Graduação em Psiquiatria e Ciências do Comportamento, Faculdade de Medicina - UFRGS, Porto Alegre, RS Brazil; 3 Programa de Pós-Graduação em Avaliação e Produção de Tecnologias para o SUS - GHC, Porto Alegre, RS Brazil; 4 Programa de Pós-Graduação em Saúde Coletiva - UFRGS, Porto Alegre, RS Brazil; 5 Institute of Medical Psychology, LMU, Munich, BY Germany; 6 Visiting Professor at UFRGS/CAPES, Porto Alegre, RS Brazil

## Abstract

Quilombos are settlements originally founded by Africans and African descendants (Quilombolas) in remote parts of Brazil to escape slavery. Due to individual histories, Quilombos nowadays exhibit different states of industrialisation, making them ideal for studying the influence of electrification on daily behaviour. In a comparative approach, we aimed to understand whether and how human sleep changes with the introduction of artificial light. We investigated daily rest-activity-rhythms and sleep-patterns in the Quilombolas’ by both wrist actimetry and the Munich ChronoType Questionnaire (MCTQ; the results of these two instruments correlated highly). Seven communities (*MCTQ:* N = 213/*actimetry:* N = 125) were compared in this study. Light exposure, phase of activity, sleep timing and duration differ across communities with various levels of urbanisation and histories of access to electricity. People living without electricity and those, who acquired it only very recently on average sleep earlier than those in more urbanised communities (mid-sleep about 1 hour earlier); sleep duration tends to be longer. Our results and those of others show that use of electricity and modern lifestyles have changed sleep behaviour. To understand the consequences of these changes for health, further studies are warranted.

## Introduction

The strategies that help organisms to cope with cyclic environments (daily or seasonal) include anticipating their regular changes. Practically all organisms have therefore developed biological clocks. These temporal programmes need to run in synchrony with their cyclic environment. They secure this by actively entraining to light signals, which are the source of all rhythmic changes (temperature, resources, predators, etc.). Light and darkness are therefore the predominant entraining agent (so-called zeitgeber) that biological clocks use for entrainment^1–3^. The entrainment process results in a stable phase relationship between the internal, circadian time and the external light-dark-cycle time. This ‘phase of entrainment’ is reflected in all aspects of physiology and behaviour (e.g., body temperature, metabolism, activity/rest, wake/sleep)^4^. It depends both on how an individual’s clock responds to a zeitgeber and on how strong the zeitgeber is. In industrialised societies, the majority of people spend most of their time indoors, thereby being exposed to relatively dim light during the day and lack of darkness after sunset (due to the use of artificial light). As a result of this drastically reduced zeitgeber strength, the circadian clocks of most people delay^5^, while work schedules remain similar. People therefore routinely use alarm clocks on workdays and thereby accumulate a sleep debt, which they compensate for on weekends. This weekly structure of alternating short-early and long-late sleep is called social jetlag^6^ and is calculated as the difference between the respective mid-sleeps on work-free and workdays. Social jetlag is associated with several health issues, including depressed mood, obesity, and cardio-metabolic risk^7–10^.

While recent studies have shown that electric lighting can influence sleep timing, its impact on sleep duration is controversial^11–16^. De la Iglesia *et al*. showed that indigenous communities with access to electricity sleep later and shorter when compared to communities without artificial light in the Argentinean Gran Chaco^12^. When Amazon rubber tappers have access to electricity, their sleep duration was also shorter and their sleep onset and dim-light melatonin onset delayed^13^. On the other hand, Yetish *et al*. suggest that “modern humans” do not sleep shorter than pre-industrial societies^14^. However, this study investigated samples of hunter-gatherers that had no electricity, without comparisons to controls (e.g., similar lifestyle but access to electricity). A more recent study compared a Mozambican village with no access to electricity to a neighboring town and found that individuals in the former slept earlier but not longer^16^, whereas a small-scale agricultural society in Madagascar was reported to sleep shorter than industrial societies^17^.

Here, we report results from studying light and sleep in Quilombolas communities. Originally, Africans and African descendants founded these settlements (called Quilombos) in remote areas of Brazil to escape slavery and hide from recapture. Presently, Quilombos exist in divers geographical areas of Brazil^18^. Quilombos are especially apt for studying changes in sleep across industrialisation because they exist in all states, from rural (exposed to sunlight during the day and to actual darkness at night) to urban (predominantly working indoors with access to artificial light). The communities compared here are situated in rural areas in the south of Brazil and rely predominantly on natural light (outdoor work), contrasting the industrialised urban 24/7 society. These communities have different histories in their exposure to electricity, and therefore represent a unique population for studying how light affects behaviour without potential confounders of urban lifestyles.

## Methods

### Participants

**Figure 1:**
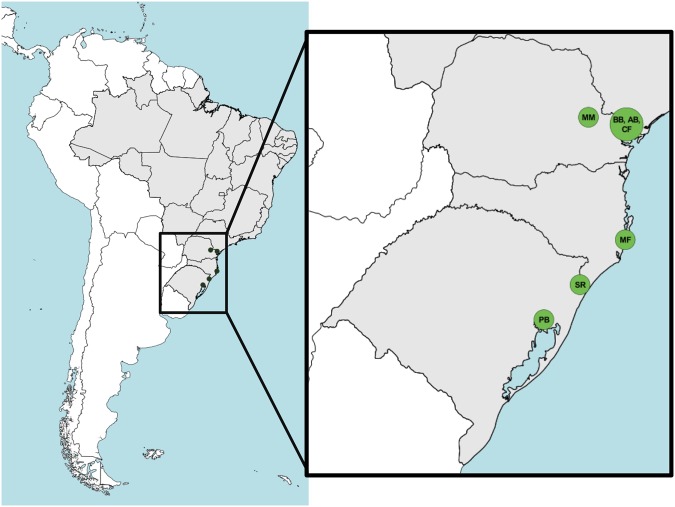
Studied Quilombos in the South of Brazil. Peixoto dos Botinhas (PB–Viamão, RS), São Roque (SR–Praia Grande, SC), Morro do Fortunato (MF–Garopaba, SC), Mamãs (MM–Castro, PR), Bombas (BB–Iporanga, SP), Areia Branca (AB–Bocaiúva do Sul, PR), Córrego do Franco (CF–Adrianópolis, PR). Maps created using Mapbox TileMill (v. 0.10.1).

Quilombolas participants (all Portuguese native speakers, *MCTQ:* N = 213/*actimetry:* N = 125) were older than 16 living in the South of Brazil distributed among seven Quilombos in nine cities and four states (Fig. 1). Data were collected between March 2012 and March 2017.

The study was approved by the Ethics Committee of Hospital de Clínicas de Porto Alegre (#11-0502, #15-0568) and was conducted in accordance with the Declaration of Helsinki. Participants gave written informed consent. When the participant was illiterate, an informed consent witnessed by another Quilombola was obtained. When the participant was younger than 18 years old, the parents also gave their consent.

The following paragraphs describe the communities and the areas where they are spread over.

#### Vale do Ribeira (PR/SP)

Vale do Ribeira comprises many traditional and local communities, including three of the seven Quilombos included in this study: Bombas, Areia Branca, and Córrego Franco. It is an area in the south of the state of São Paulo and north-east of the state of Paraná, currently with an estimated 411,500 inhabitants. It contains 21% of the remaining Atlantic forest with protected areas; it is home for a large number and exceptionally varied endemic species, but is economically poor. The climate is humid subtropical. Evidence suggests that it was populated by native south Americans long before Europeans occupied it in the 16^th^ and 17^th^ century, predominantly using African labour to mine for gold. The first city was founded in 1567 (Iporanga). After the mines were abandoned the region became agricultural. In the transition, many former slaves claimed lands and developed an agriculture focused on the food market (for both local consumption and trade with other regions). Rice cultivation is an example: its cycle started in the end of the 17^th^ century, and it was intensely commercialized to other provinces of the Empire of Brazil until mid 19^th^ century. Many former slaves settled into the woods, became small farmers and gave rise to communities in the area^19^.

Bombas (BB–Iporanga, SP): Bombas area is located in a natural reserve in the heart of the Atlantic forest, near to the small town Iporanga in the south of the state of São Paulo. The area was first occupied in the 19^th^ century; the historical reference is of slaves who fled and families that were forced to leave their lands by a lead mining company. Iporanga’s history is marked by gold mining and rice cultivation. Bombas is situated in a conservation area, which prevented the construction of roads until today and thus, the communities remained isolated, accessible only on a trail over hills covered with woods. The community is divided in two main parts: Bombas de Baixo (≈5 km from the main road) and Bombas de Cima (≈10 km from the main road) neither of which have yet been connected to the electricity grid. It takes 2–3 hours to reach the first houses of Bombas de Baixo on foot. This Quilombo is not a closed village, but houses are dispersed over an area of approximately 32 km^2^. Nevertheless, they have a strong sense of community. Quilombolas report to visit the city on average once to twice a month and some of them spend the night there. They practice an itinerant form of agriculture known as ‘coivara’ (i.e., opening a clearing in the forest during the dry season by cutting down trees and hoeing and burning it to enrich nutrients before planting). This coivara is often used in forest areas and presents an essentially subsistence character, even though surpluses might be sold (mainly to buy clothes, construction materials or salt in the city). Besides subsistence agriculture and animal breeding, the community survives from social donations^18^. Bombas Quilombolas lead a pre-industrial life. Work activities are organised according to weather. Their definition of workdays vs. free days does not necessarily reflect the one of industrialised societies. Before retreating to bed, they use oil/gas lamps and sit close to clay ovens to eat and talk. Their houses are made of mud and/or wood and people sleep indoors on beds with mattresses. Light at night is restricted to oil/gas lamps used mainly while dinner is being prepared while the rest of the house remains in darkness. Flashlights are used but mainly to move from one room to another and for going outdoors. Data were collected in 2016–2017.

Areia Branca (AB–Bocaiúva do Sul, PR): Areia Branca is part of the municipality of Bocaiúva do Sul, in the state of Paraná, about 115 km from this large city, and 50 km from the small town Barra do Turvo. According to the collective memory of the community’s members, a recaptured slave established the settlement when given some land, which can now be accessed on dirt roads. The community installed a photovoltaic system in 2014 (our data were collected between August–December 2016), which provided electricity when weather permitted. Some members of the community have already lived in the city. The community lives mostly from agriculture. Recently, with support from the government, they also got a community kitchen abling the production of jams and bananas/manioc chips to be sold in nearby towns^20,21^. Their houses are made wood and/or concrete. Although they have bulbs indoors to light their night, individuals reported they still run out of electricity after extend periods of overcast days. Individuals usually have televisions at home, only a few have cell phones, internet signal was not available when data were collected, in 2016.

Córrego do Franco (CF–Adrianópolis, PR): Córrego do Franco is part of the municipality of Adrianópolis, in Paraná, about 124 km from the next large city and 10 km from the nearest small town, Barra do Turvo. The community has lived in the region for about 250 years. According to the official community’s anthropologic Report, the inhabitants’ life is tightly connected to subsistence agriculture, with a daily work routine following climate rhythms. However, they often work for a salary in the plantations of large landowners or in the city to supplement their income^20,22^. Members of the community report to have had electricity for around 20 years (before our still ongoing data collection was started in 2012). People live in wood/concrete houses (many of which permanently under construction), and people’s houses are relatively close to each other when compared to the two communities described above. They have light bulbs, televisions and some participants already had access to internet when data were collected, in 2016.

#### Praia Grande (SC)

The Quilombola community São Roque, from this municipality was included in this study. Praia Grande is a municipality in the south of Santa Catarina (≈7,364 inhabitants^23^. Despite meaning “large beach”, the town is landlocked and is near to the canyons. The climate is temperate with a considerable forest reserve. It is predominantly an agricultural economy but tourism is a growing business.

São Roque (SR–Praia Grande, SC): This Quilombolas in Praia Grande pursue agriculture and its headquarters is located about 20 km from the city centre. Slaves were usually forced to do domestic and manual works. When sent to do field work in or to deliver goods to other locations, some took to refuge when travelling through remote regions between the valleys and caves that line the hillsides and formed supporting networks in the 19^th^ century. Agriculture has been the main mean of survival, and currently, maize, beans, bananas and manioc are among the main harvests. The other main sources of income are working in domestic and agricultural jobs, and retirement^24–27^. São Roque is located in a protected area (national park), and is also engaged in touristic services. The community reports to have had electricity for about 15 years, and except for one individual, they had light bulbs at home. Most houses are made of either wood or concrete (the younger generations seek to build their houses nearer to the city, using concrete). Data were collected in 2013–2014.

#### Castro (PR)

Castro comprises four Quilombos, of which one, Mamãs, was included in this study. Castro is the third largest municipality in terms of area in the state of Paraná (estimated 71,501 inhabitants^23^), founded in the 18^th^ century on the trail connecting Viamão in Rio Grande do Sul with Sorocaba in São Paulo. The climate is subtropical, and the economy is based on agriculture and dairy farming developed by Dutch colonies^28^.

Mamãs (MM–Castro, PR): Mamãs is located about 60 km from the city centre of Castro. The community is divided in many family centres living up to 70 km distant from each other, and some of the families are located in the neighbour municipality of Cerro Azul. The history of this dispersed community goes back to a farm that was owned by Carmelite Fathers in 1749. The farm was abandoned and taken care for about a hundred years by the African descendants who were previously forced to work there. After about a century of freedom, in 1864, these descendants did not agree when the priests negotiated the farm and they were sold to a company: the new landowners wanted to take them to São Paulo as slaves. After being defeated by the military force in an uprising, the ones who could, fled to different areas forming the communities Serra do Apon e Mamãs^20^. The Quilombolas in Castro live from agriculture and, as in Córrego do Franco, they often work in the plantations of large landowners or in the city to complement their income. People from Mamãs reported they have had electricity for around 20 years, though some got it later, about 8 years ago. Most houses are made of wood. Mobile signal was poor to inexistent when data were collected and Internet signal was also unavailable. The majority but not all people had televisions and they did not have smartphones. Data were collected in 2014–2016.

#### Garopaba (SC)

Garopaba comprises two Quilombolos, of which one, Morro do Fortunato, was included in this study. Garopaba is a Brazilian municipality on the southern coast of the state of Santa Catarina. Its estimated 22,082 inhabitants^23^ are mainly engaged in tourism, construction, fishing and subsistence agriculture. The area was colonised by Portuguese from the Azores Islands in the 17^th^ century and still retains many traces of the Azorean culture.

Morro do Fortunato (MF–Garopaba, SC): Morro do Fortunato is located in Garopaba, about 8 km from the city centre. Their territory is very close to the seaside and it is told that the lands were given to Fortunato, the son of a white landowner and his slave. These lands were deep in the woods because he did not want Fortunato, with marked genetic characteristics of European and African miscegenation (e.g. dark skin and blue eyes), to draw attention from the ‘white community’. According to one of the leaders, who was 55 years old in 2013, he is Fortunato’s great grandson. This Quilombo, therefore, emerged as a place of making miscegenation invisible to the white society and, following that, marriages with African descendants from other communities, and consanguine marriages designed its constitution. The inhabitants lived for decades from subsistence agriculture and breeding and many of them still develop agricultural practices in rural areas, ranging from breeding livestock to growing sugar cane^29,30^. Women also produce jam to sell locally and the community is engaged in tourism. Inhabitants and the electricity company report electricity was installed 30 years ago. People in MF have light bulbs and television at home, and street lighting. Most houses are made of concrete. Only a few people have access to Internet at home. Data were collected in 2013–2014.

#### Viamão (RS)

In Viamão, a Quilombola community (Peixoto dos Botinhas) was included in this study. Viamão is a city in the metropolitan region of the state of Rio Grande do Sul (estimated population: 239,384^23^) founded in 1741. Important commercial routes began where the municipality is located and it is the region where the first cattle ranches were established. Its economy is still based in farming and services.

Peixoto dos Botinhas (PB–Viamão, RS): This Quilombo is located in the municipality of Viamão about 86 km from the city centre, close to a highway, in a rural area. Public transportation service is available throughout the day, and only few families live remote. The community origins refer to two African ancestors, who occupied vacant land after disembarking on a nearby lagoon (Lagoa dos Patos) and built ranches. The communities occupied lands isolated and considered marginal then, but that today are valued by the agribusiness for its location, with great potential for rice production and cattle breeding^31^. In Peixoto dos Botinhas, people are not only engaged in agriculture, but many inhabitants are also personal service workers. According to the responsible grid company, electricity was brought to the first families around 1977. People reported adhesion to the use of electricity to be a slow process. In general, people did not have smartphones when data were collected, but had light bulbs and televisions at home. Houses were made of either wood or concrete. Data were collected in 2012–2013.

### Procedure

Communities were selected based on geographical localisation, and history of access to electricity. After first contact with Quilombo organizations and the approval of the community leaders, meetings were organised at the Quilombo’s association headquarters (located in the community territory). After participants gave their informed consent, they were interviewed and provided with the respective instruments (either right after the meeting or at their homes).

### Instruments

Questionnaires were adapted to the Quilombolas cultural context and interviewers were trained to inquire in a standardised way about sleep-wake behaviour and average natural light exposure using the Munich ChronoType Questionnaire (MCTQ).

#### Demographic characteristics

Demographic characteristics were collected using a standard questionnaire^32^. Participants were asked about their age, educational level, occupation, as well as drinking and smoking habits. They were also asked about medical history (whether they present or not any chronic disease) and whether they take any medication.

#### Munich ChronoType Questionnaire (MCTQ)

The Brazilian Portuguese version of the Munich Chronotype Questionnaire (MCTQ) was applied to assess sleep-wake behaviour and self-reported natural light exposure on work and work-free days. It asks, separately for workdays and work-free days, at what time people go to bed and are ready to sleep, how long it takes them to fall asleep, at what time they wake up and get up and if they use an alarm clock. A number of variables can be derived from MCTQ data, including sleep duration, chronotype (midpoint between sleep onset and sleep offset on work-free days, corrected for oversleeping if individuals sleep longer on work-free days than on work days, MSFsc) and social jet lag (difference between mid-sleep on work and work-free days^6,33^). Self-reported outdoor light exposure is also assessed separately for work and work-free days using specific questions. 215 participants filled out the MCTQ, out of which data from two could not be used (overly irregular patterns reported). Both the English version of the questionnaire (*English MCTQ core* + *time spent outdoors question from MCTQ full*) and information on the calculation of the variables can be found at: http://thewep.org/documentations/mctq.

#### Actimetry

For actimetry analyses, the inclusion criteria were: continuous actimeter use for at least 7 days. Days were not included in the calculation of activity phase markers and light exposure when more than 4 hours were missing. Missing episodes were identified as stretches of at least 5–10 consecutive bins (50–100 min) of no activity and were excluded from the analysis. Wrist actimeters (Actiwatch 2: Philips Respironics, ActTrust: Condor, Daqtomter: Daqtix) were distributed to 148 participants, out of which data from 125 (84%) could be used considering the excluding criteria aforementioned. Data were averaged into 10-min bins for analyses. Actimeters were shown not to differ in sleep detection (Fig. S1A,B). Light intensity data (daily averages of light exposure during photoperiod and daily averages of light exposure after dusk) were normalised using the correlation slope equation from data collected over 14 days using both actimeters (Actiwatch 2 and ActTrust: 1657 bins of 10 min, representing 276 hours of recordings) at the same time (Fig. S1C). The only two light sensors used from Daqtix did not work and these subjects could not be included in light analyses. Data from Bombas, Areia Branca and Córrego do Franco were collected using ActTrust. Data from São Roque, Morro do Fortunato and Peixoto dos Botinhas were collected using Actiwatch 2. Data from Mamãs were collected using both brands. Data from two subjects from Areia Branca were collected using Daqtix.

The software ChronoSapiens^33^ was used to asses activity phase markers (i.e., centre of gravity–acrophase–of the first harmonic fit), sleep onset, sleep end, mid-sleep, sleep duration, and light exposure patterns (average light exposure during photoperiod and after dusk). For all variables derived from actimetry, group central tendency measures were calculated using subjects’ daily averages. As previously described^33^, sleep bouts in activity records were identified using stretches of relative immobility; bins with activity counts below 20% of the 24-hr centered moving average were classified as potential sleep; this retrieval was then filtered for sleep bouts with durations of at least 30 min and consolidated into longer bouts based on correlations with produced test series of different lengths. For this study, bouts with durations of 3–12 hours were included. Bouts interrupted for less than 2 h were combined (second bout length: 120–540 min). Bout-lengths were considered as sleep durations. When bouts were fused, sleep duration was calculated as the sum of their lengths, sleep onset was taken from the first bout while sleep offset was taken from the second. Mid-sleep was calculated as midpoint between sleep onset and offset. Since we focused on sleep at night, episodes that began after 8 am and ended before 3 am were checked for and manually excluded.

### Data Analysis

Shapiro-Wilk was used to test continuous variables for normality. To validate the use of the MCTQ in Quilombolas communities, the correlation between data from actimetry and MCTQ was tested using Pearson. Light exposure data were analysed using Kruskal-Wallis followed by Dunn’s test adjusted by Bonferroni correction. Activity phase and sleep patterns of communities were compared using ANOVA, followed by Tukey. Statistical significance was set at p < 0.05. SPSS 24 and GraphPad Prism 6 were used for statistical analysis. Colour codes for all graphs were RGB calculated using a Geographical Isolation Index. This index was calculated by multiplying the urbanisation rate of the municipality by 1 if there was no access to vehicles, 3 if public transport was available 1−2x/day, 5 if public transport was available more than 2x/day or many inhabitants have cars (Geographical Isolation, Table 1). The fraction of red, green and blue were then calculated proportionally to this index, with the greenest being the community more isolated, and the reddest the least.

**Table 1:** Characteristics of each Quilombo studied.

	Municipality	Urbanisation rate (%)^a^	Difficulty of access^b^	Geographical Isolation^c^	Electricity history^d^	Coordinates (decimal degrees)
Bombas (BB)	Iporanga (SP)	55.8	1	55.85	No electricity^*^	48.6W 24.6S
Areia Branca (AB)	Bocaiúva do Sul (PR)	46.7	3	140.02	2 yrs^*^	48.6W 25.0S
São Roque (SR)	Praia Grande (SC)	59.1	3	177.39	15 yrs^*^	50.0W 29.2S
Córrego do Franco (CF)	Adrianópolis (PR)	32.3	5	161.54	>20 yrs^*^	48.6W 24.8S
Mamãs (MM)	Castro (PR)	73.4	3	220.32	20 yrs/8 yrs^*^	49.7W 24.8S
Morro do Fortunato (MF)	Garopaba (SC)	84.5	5	422.32	30 yrs^*#^	48.6W 28.0S
Peixoto dos Botinhas (PB)	Viamão (RS)	94.0	5	469.84	>30 yrs^#^	51.0W 30.1S

^a^Referring to the entire municipality; source: last census of the Brazilian Institute of Geography and Statistics^49^.

^b^Subjective classification: 1 no access to vehicles; 2 no public transport; 3 public transport 1−2x/day (far from the city centre); 4 (near to the city centre); 5 public transport more than 2x/day or many inhabitants have cars.

^c^Calculated by multiplying urbanisation rate by difficulty of access.

^d^*Reported by inhabitants; ^#^reported by electric company.

## Results

### Population characterisation

The Quilombola population (55% women) had an age range between 16 and 92 (mean: 45.2 ± 18.2 years; see demographic characteristics by community in Table 2) and lived mostly in rural areas. Most commonly reported occupations were farming (35%) and housekeeping (21%). 12% reported to be retired and 8% unemployed. The vast majority of subjects in Bombas (BB), Areia Branca (AB), São Roque (SR), Córrego do Franco (CF) and Mamãs (MM) were farmers and/or housekeepers. A small portion reported to be retired, unemployed or work as personal service/health workers. In Morro do Fortunato (MF) and Peixoto dos Botinhas (PB), fewer participants reported to be farmers and a considerable proportion of subjects works in elementary occupations/as personal service or sales workers (Table S1).

**Table 2:** Characteristics of the Quilombolas sample (*MCTQ:* N = 213/*actimetry:* N = 125).

	N MCTQ/acti	Age (mean ± SD)	Sex (%F)	Housekeeper or Farmer (%)	Regular work schedule (%)	Alarm clock usage (%)	Alarm clock need (%)	Season acti (S; W)
Bombas (BB)	29/27	33 ± 15/31 ± 14	48/52	93/93	3/4	3/4	0/0	5; 22
Areia Branca (AB)	13/14	55 ± 12/54 ± 12	46/50	92/93	38/36	0/0	0/0	2; 12
São Roque (SR)	25/18	44 ± 16/42 ± 15	44/50	64/61	56/60	28/33	8/13	11; 7
Córrego do Franco (CF)	16/11	33 ± 10/33 ± 11	50/54	69/64	56/56	44/56	6/11	5; 6
Mamãs (MM)	29/19	41 ± 19/43 ± 21	62/68	76/79	38/32	28/26	14/16	11; 8
Morro do Fortunato (MF)	60/18	46 ± 19/61 ± 19	55/72	30/22	68/47	45/29	34/17	4; 14
Peixoto dos Botinhas (PB)	41/18	57 ± 14/59 ± 15	63/61	29/39	68/67	15/11	8/6	8; 10

Averages from subjects who filled the MCTQ/wore the actimeter appropriately.

Regular work schedule: reported (MCTQ).

Season: actimetry data-summer; winter based on start of data collection.

### Validation of the MCTQ in Quilombos

Subjective data from the MCTQ correlated highly with data calculated from actigraphy data (Figure S2).

### Light exposure

**Figure 2:**
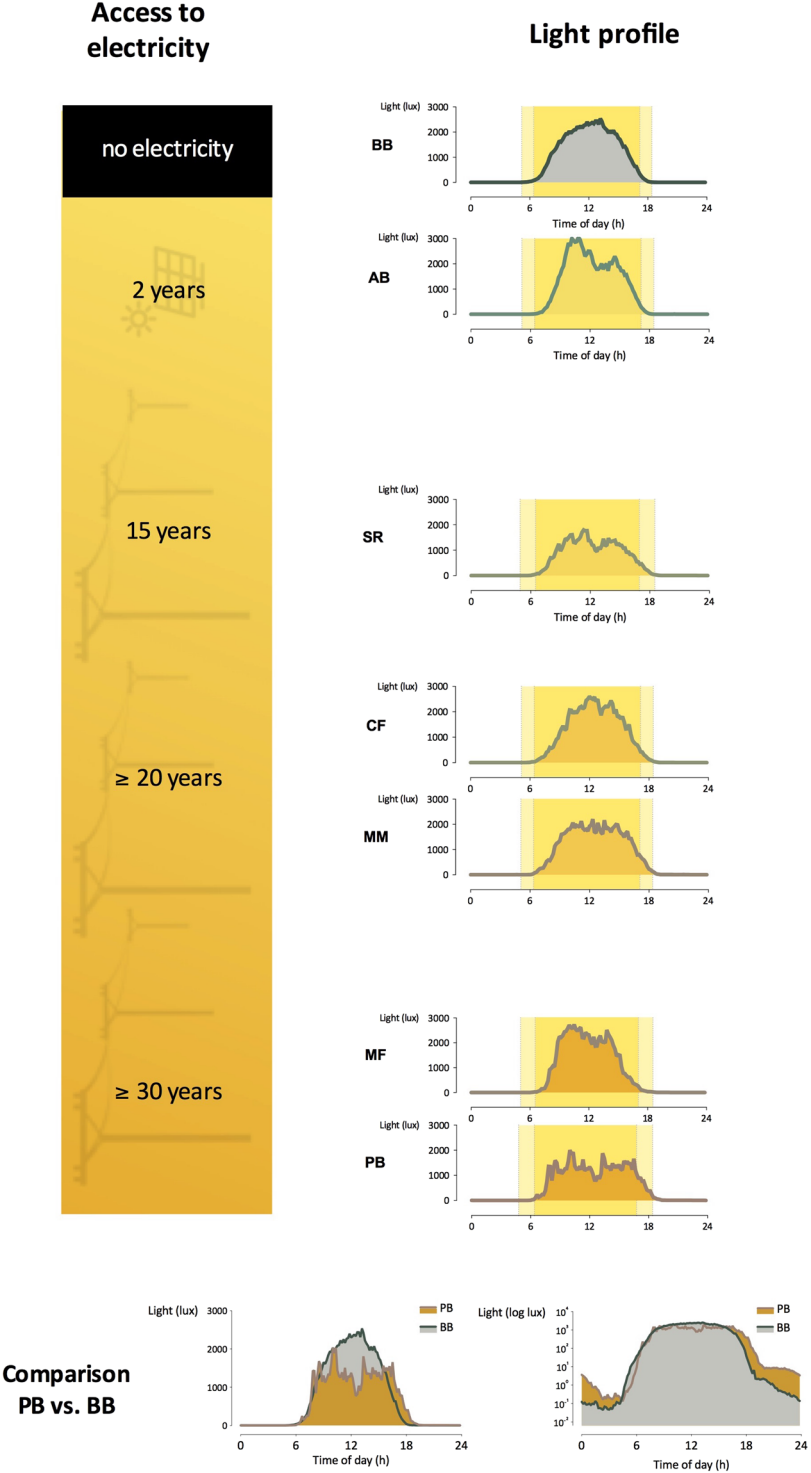
Average light profiles: each graph shows the average light profile of a community, calculated using individuals actimetry data. A comparison between the extremes, Bombas (BB) and Peixoto dos Botinhas (PB) can be seen at the bottom, being the graph to the right log scaled. Backgrounds represent photoperiod length (on the longest and shortest day). n = 11–27. BB: Bombas, AB: Areia Branca, SR: São Roque, CF: Córrego do Franco, MM: Mamãs, MF: Morro do Fortunato, PB: Peixoto dos Botinhas.

**Figure 3:**
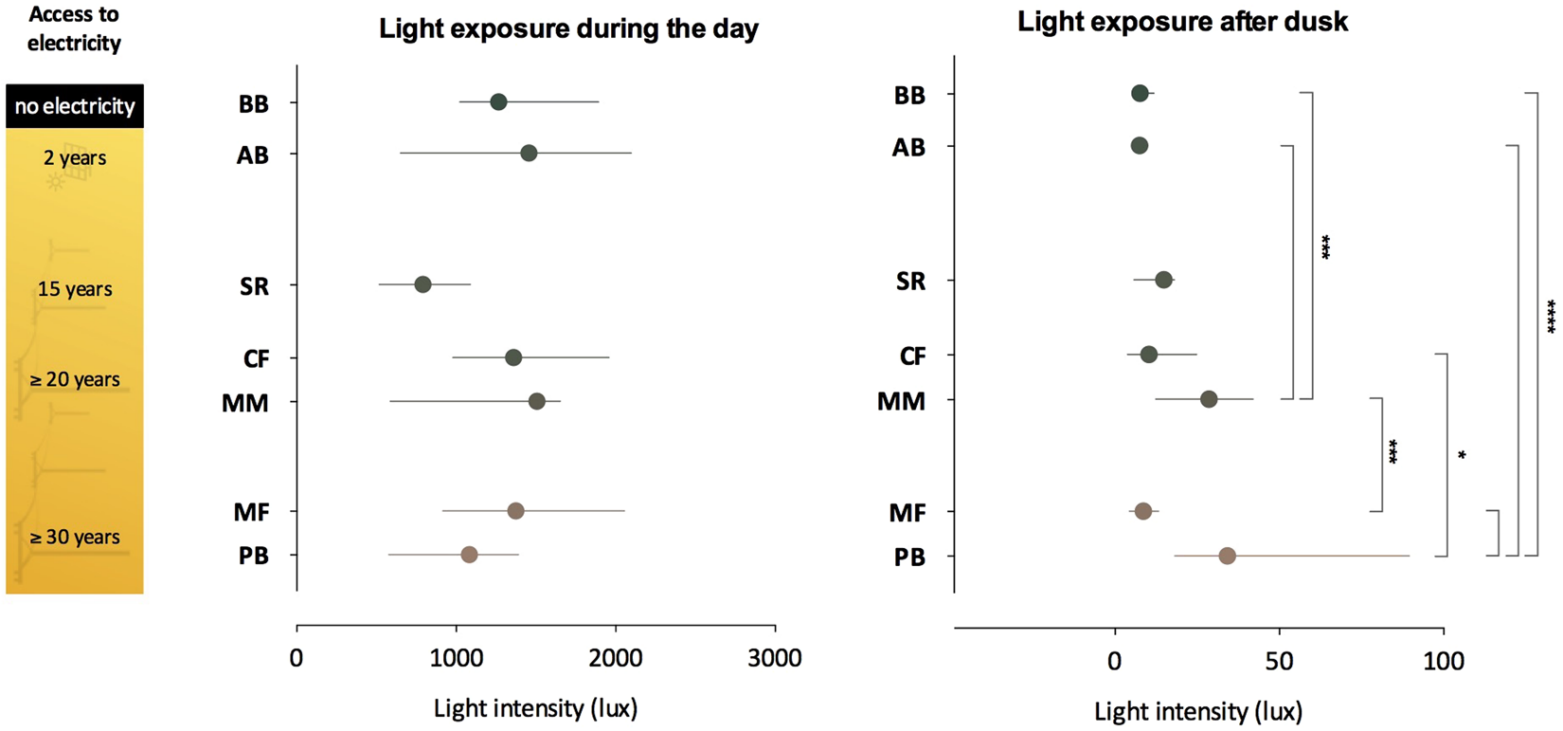
Light exposure: dots represent the median, whiskers represent interquartile ranges of light exposure during photoperiod (Kruskal-Wallis, χ²(6) = 11.95, p = 0.06) and after dusk (Kruskal-Wallis, χ² (6) = 50.96, p < 0.0001). Dots are colour coded according to the geographical isolation index. The greener the bar, the more geographically isolated the community. Dunn’s test adjusted p-values: ^*^p < 0.05; ^**^p < 0.01; ^***^p < 0.001; ^****^p < 0.0001). n = 11–27. BB: Bombas, AB: Areia Branca, SR: São Roque, CF: Córrego do Franco, MM: Mamãs, MF: Morro do Fortunato, PB: Peixoto dos Botinhas.

Average light profiles of each community can be seen in Fig. 2. Figure 3 shows the average light exposure for each community during photoperiod and from dusk to midnight. No statistical difference between groups but a trend (Kruskal-Wallis, χ²(6) = 11.95, p = 0.06) was observed in exposure to light during photoperiod. People in PB and MM were on average exposed to higher levels of light after dusk than BB, AB and MM.

### Activity and sleep phase

**Figure 4:**
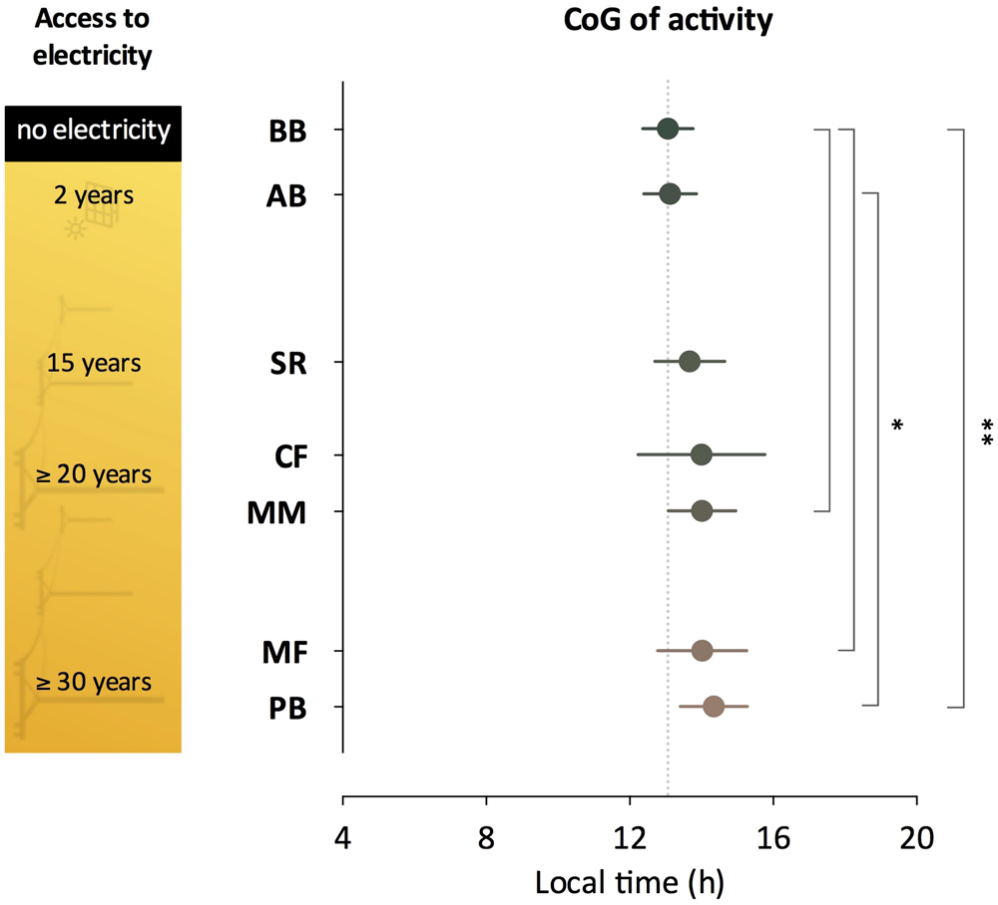
Centre of Gravity of Activity: dots represent the mean, whiskers represent the standard deviation. Dots are colour coded according to the geographical isolation index. The greener the bar, the more geographically isolated the community. The dotted line marks BB mean as a reference. ANOVA, F_(6,118)_ = 4.40, p < 0.001. Tukey’s adjusted p-values: ^*^p < 0.05; ^**^p < 0.01. n = 11–27. BB: Bombas, AB: Areia Branca, SR: São Roque, CF: Córrego do Franco, MM: Mamãs, MF: Morro do Fortunato, PB: Peixoto dos Botinhas.

The centre of gravity of activity (CoG_act_) delayed systematically with longer artificial light history from BB to PB (Fig. 4).

**Figure 5:**
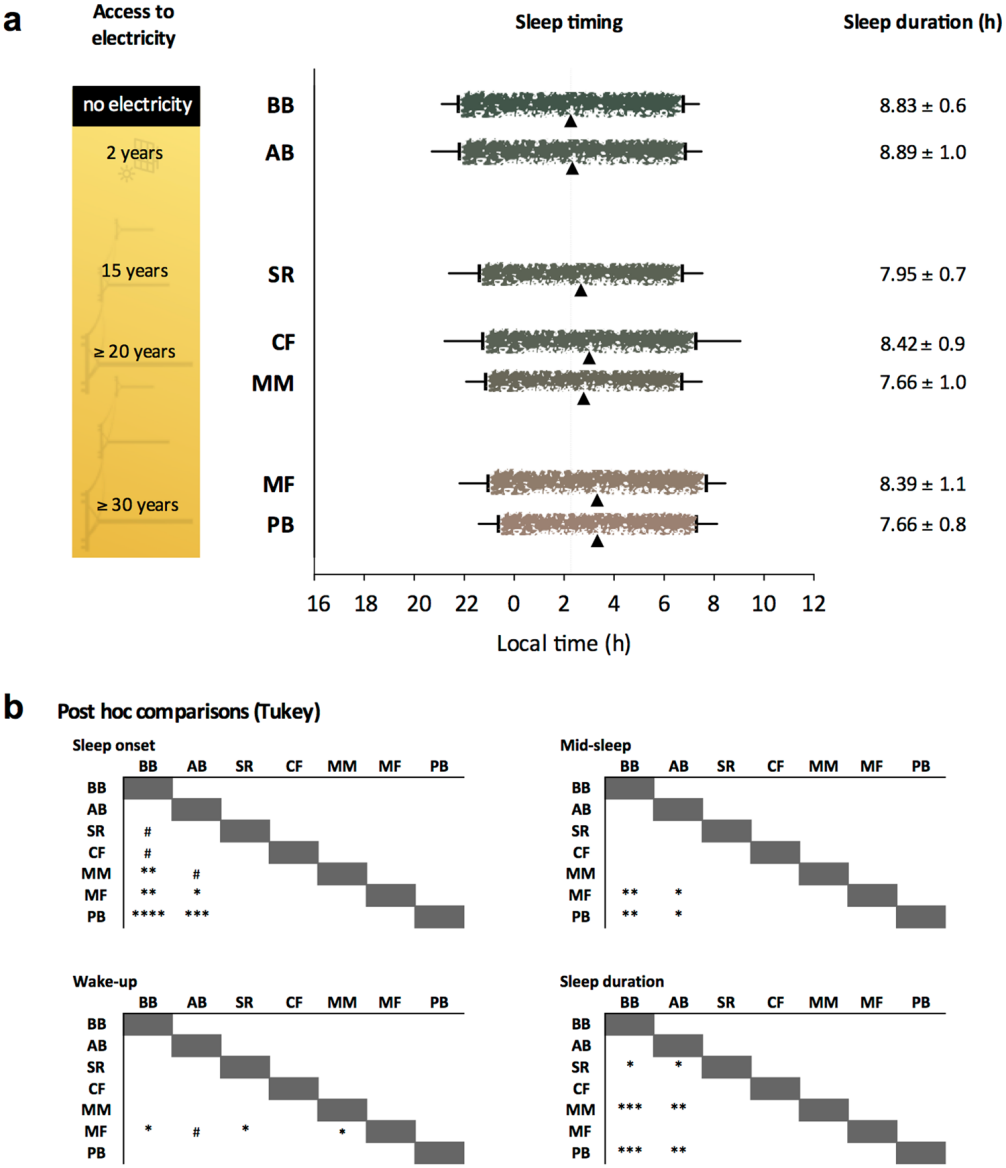
Sleep timing and duration: (**a)** ‘Brushed’ lines represent average sleep episodes calculated from actimetry data. Horizontal whiskers represent standard deviation and triangles mid-sleep. Episodes are colour-coded according to the geographical isolation index. The greener the bar, the more geographically isolated the community. The dotted line marks BB mid-sleep mean as a reference. Sleep onset: ANOVA F_(6,118)_ = 6.88, p < 0.0001, mid-sleep: ANOVA F_(6,118)_ = 5.09, p < 0.001, wake-up: ANOVA F_(6,118)_ = 3.36, p < 0.01, sleep duration: ANOVA F_(6,118)_ = 6.54, p < 0.0001. (**b**) Tukey’s adjusted p-values: ^*^p < 0.05; ^**^p < 0.01; ^***^p < 0.001; ^****^p < 0.0001, ^#^p < 0.15). n = 11–27. BB: Bombas, AB: Areia Branca, SR: São Roque, CF: Córrego do Franco, MM: Mamãs, MF: Morro do Fortunato, PB: Peixoto dos Botinhas.

Based on the sleep-assessment of the actimetry data, sleep onset in PB and MF was statistically later than in BB and AB, and in MM than in BB. The mid-sleep also occurred later in PB and MF than in BB and AB. MF presented later wake up times than BB, SR and MM. PB, SR and MM slept shorter than AB and BB (Fig. 5).

Considering that data were collected in different seasons, we performed the same analyses using only data from March to September and retrieved similar results (Table S2). We also found similar results comparing communities’ reported sleep times on work-free days from the MCTQ (Table S2).

Average light exposure was significantly correlated with sleep onset and duration (calculated from actigraphy), both during photoperiod (Spearman, *onset:* r = −0.28, p < 0.01; *onset - no Daylight Saving Time change:* r = −0.35, p < 0.001; *duration:* r = 0.18, p < 0.05) and after dusk (Spearman, *onset:* r = 0.30, p < 0.01; *onset - no Daylight Saving Time change:* r = 0.21, p < 0.05; *duration:* r = −0.23, p < 0.05). Zeitgeber strength (ratio of light exposure during photoperiod to light exposure after dusk) also correlated with sleep onset and duration (Spearman, *onset:* r = −0.46, p < 0.001; *onset - no Daylight Saving Time change:* r = −0.38, p < 0.001; *duration:* r = 0.37, p < 0.001). Alarm clock usage was also associated with sleep duration (*use alarm:* 8.33 h ± 1.01, *do not use:* 7.88 h ± 0.86. Student’s t test, t_116_ = 1.95, p = 0.05).

A hierarchical multiple linear regression was performed to assess significant predictors of midpoint of sleep and sleep duration and control for effects of age and sex. In both models, the effect of ‘community’ was significant when controlling for age and sex. When season of data collection and exposure to light during photoperiod were added, they were also significant predictors. Having regular work schedules was not included in favour of a parsimonious model for it was not a significant predictor of sleep timing or duration. These results are detailed in Tables S3 and S4.

## Discussion

The primary findings of this study corroborate the notion that phase of activity and sleep changed as a result of electricity usage and other modern lifestyles. Communities, which have not yet or very recently acquired electricity, sleep earlier than those which have been connected to the grid long ago. These results are similar using different assessment methods (subjective questionnaires versus objective light and activity measurements) and both correlate highly. Even the Quilombolas who live without electricity have a clear concept of clock time. Many of them use clocks to communicate with the outside world, but not necessarily to organise their day. It was therefore possible to ask them the questions of the MCTQ and get meaningful answers based on the subjects’ concept of local time. From knowledge about circadian entrainment^34^ and experimental studies^35^, our results on sleep timing were predictable due to varying zeitgeber strength. The combination of high daylight exposure and low light at night generates a strong zeitgeber signal while indoor work and artificial light after sunset weaken the signal. In general, the stronger the zeitgeber, the earlier the phase of entrainment (chronotype). Due to the weak zeitgebers in industrialised societies, most clocks have delayed while social schedules have remained relatively unchanged. People therefore accumulate sleep debt over the workweek determining the amount of social jetlag they suffer from^9^. Even Peixoto dos Botinhas (PB), according to the Geographical Isolation Index the closest to an urbanised community, is still a rural one, showing a considerably high average light exposure during the day. Despite the mostly rural lifestyles in the different communities they did show differences in light exposure, and light exposure during the day and at night significantly correlated with sleep timing.

Sleep duration also differed between communities. Three of the five communities that had electricity for more than a decade (SR, MM and PB) sleep shorter than those with no or more recent access to electricity (BB and AB). The other two long-term electricity communities (MF and CF) did sleep 25 to 30 min shorter than BB/AB, but the difference did not reach significance. MF also had later wake-up times and we have collected actimetry data mostly from retired participants. Although the human circadian timing is mainly influenced by light, sleep is additionally influenced by many other factors related to urbanisation and life routines, which may explain the heterogeneous reports about sleep duration in the literature^12–14,16,17^. An important factor that impacts sleep is globalization: social interaction, commercial activities and work responsibilities are not local anymore, but often virtually connected across time zones. Although they might communicate using mobiles (rarely smartphones), the Quilombolas described here have no access to the Internet or were connected only very recently. Sleeping arrangements and strain of daytime work are certainly important factors to be considered. Despite houses (e.g., made from mud/clay, wood, or concrete) and environmental noise varying in the Quilombos, people sleep indoors and on beds with mattresses in all communities we have visited. High physical work (e.g. rubber tappers in Acre, Brazil) was associated with lower sleep quality^36^; physical inactivity was likewise reported to be a predictor of sleep complaints and depression^37^. While physical exercise is recommended to prevent or treat sleep disorders, the interrelationship between these two factors is not yet fully understood^38^. It remains to be tested in future studies how the levels of physical activity during work and leisure in Quilombos may be a factor contributing to the differences seen in sleep behaviour.

In this study, all communities mainly live from farming, but may vary in the way they see productivity and subordinate it to time. In the words of the leader of the community that has no electricity: “In the city, it is easier to get tired. It is a matter of time. During the day, everything is as soon as the clock tells. Here we work by solar time”. In the US, work is the main activity sleep is exchanged for and findings suggest that interventions aiming to increase sleep duration should focus on delaying start times of work or making them more flexible^39^. Adolescents with electric light were seen to sleep later than adolescents without electricity at home, but only those, who attended morning classes and had electricity showed a reduction of night sleep duration^15^. In line with our study presenting a relatively long sleep in Quilombos, longer sleep and lower prevalence of short sleep duration have been reported in farmers (large cross-sectional studies in China and the United States)^40,41^.

Less than 10% of people from the community that has no electricity (BB) report to have regular work schedules, whereas in PB (the less isolated community) more than half do. Differently from the other communities, people in BB and AB, the community that has had electricity for only two years, do not use alarm clocks. Despite the percentage of people, who report the usage of an alarm clock being low in all Quilombolas communities, those who use alarm clocks sleep shorter than those who do not. Early wake up might still be culturally associated to success and productivity in some Quilombos even if to a lesser degree. Supporting this rationale, a rural community in the South Brazil was shown to present similar sleep duration than an urban one^32^ and in the Brazilian Southeast, a rural population with conservative lifestyles also showed relatively earlier wake up and bed times^42^ than what was reported in other studies in rural areas. These findings stress the importance of raising general awareness of the consequences of insufficient sleep, since whether or not sleep duration has substantially changed from pre-electricity era to present days, sleep deprivation have well-known health consequences^43–45^.

Some limitations of the study are noteworthy. The fact that we collected data over different seasons could have influenced our results^46^. However, the differences in sleep timing remained similar when comparing only data collected in the winter. Our study was conducted over 4 years, allowing for the first time to study many communities that are engaged in similar daytime activities, but differ in urbanisation and access to electricity. The differences in mean age between the communities could represent another confounder considering that elder people usually sleep shorter and earlier^47,48^. However, results were similar when controlling for age and sex. Light exposure measured at the wrist may not reflect the levels received by the eye, photoreceptors sensitivity was not taken into account and subtler differences might not have been detected using actimetry. Still, light intensities should vary similarly at both the eye and the wrist.

Studying human behaviours is challenging because they are typically complex and subject to many environmental factors. However, the communities here analysed are considerably similar regarding work activities, cultural background, social organisation and they live at relatively near geographical locations with rather similar photoperiods and weather conditions (Table S5), allowing us to draw important conclusions regarding conceivable changes brought by electricity. Supporting previous studies, our findings indicate that access to electricity might have brought changes to sleep patterns. Further studies in these communities might help us to recognise the consequences of these changes for health and propose ways of minimising them.

## Electronic supplementary material

Supplementary Information
