# Supplementary material for: Sleep and light exposure across different levels of urbanisation in Brazilian communities
Source: Sci Rep. 2018 Jul 30;8:11389. doi: 10.1038/s41598-018-29494-4 (PMC6065379; doi:10.1038/s41598-018-29494-4)
Supplement: Supplementary file 1 — Supplementary Information [file 41598_2018_29494_MOESM1_ESM.docx]

**Sleep and light exposure across different levels of urbanisation in Brazilian communities**

Luísa K. Pilz, Rosa Levandovski, Melissa A. B. Oliveira, Maria Paz Hidalgo, Till Roenneberg

Supplemental Material

| TABLE S1: Quilombolas occupation: MCTQ and actimetry samples (total %) | | | | | | | | | | | | |  |  |
| --- | --- | --- | --- | --- | --- | --- | --- | --- | --- | --- | --- | --- | --- | --- |
|  |  | Farmer | Housekeeper | Farmer AND  housekeeper | Retired | Elementary occupations^*^ and personal service workers | Unemployed | Sales workers | Personal care and  health workers | Teacher | Student | Craft and related  trade workers | Machine operators (drivers) | Not reported |
| Bombas (BB) | MCTQ | 72.4 | 17.2 | 3.4 | 3.4 | 3.4 |  |  |  |  |  |  |  |  |
|  | Actimetry | 70.4 | 18.5 | 3.7 |  | 3.7 |  |  |  |  |  |  |  | 3.7 |
| Areia Branca (AB) | MCTQ | 69.2 | - | 23.1 | 7.7 |  |  |  |  |  |  |  |  |  |
|  | Actimetry | 71.4 | - | 21.4 | 7.1 |  |  |  |  |  |  |  |  |  |
| São Roque (SR) | MCTQ | 44.0 | 20.0 |  | 8.0 | 8.0 |  |  |  |  | 8.0 | 8.0 |  | 4.0 |
|  | Actimetry | 38.9 | 22.2 |  |  | 5.6 |  |  |  |  | 5.6 | 11.2 |  | 16.7 |
| Córrego do Franco (CF) | MCTQ | 37.5 | 25 | 6.3 | 6.3 | 12.6 | 6.3 |  |  |  | 6.3 |  |  |  |
|  | Actimetry | 36.4 | 18.2 |  | 9.1 | 9.1 |  |  |  |  | 9.1 |  |  | 18.2 |
| Mamãs (MM) | MCTQ | 58.6 | 10.3 | 6.8 | 10.3 | 6.8 |  |  | 6.8 |  |  |  |  |  |
|  | Actimetry | 57.9 | 10.5 | 10.5 | 10.5 |  |  |  | 10.5 |  |  |  |  |  |
| Morro do Fortunato (MF) | MCTQ | 10 | 20 |  | 21.7 | 28.5 | 1.7 | 1.7 | 1.7 |  | 3.3 | 3.3 | 3.3 | 5.0 |
|  | Actimetry | 5.6 | 16.7 |  | 44.4 | 11.2 |  | 5.6 |  |  |  |  |  | 16.7 |
| Peixoto dos Botinhas (PB) | MCTQ |  | 29.3 |  | 17.1 | 31.6 | 7.3 | 9.8 |  | 2.2 |  | 2.2 |  |  |
|  | Actimetry |  | 38.3 |  | 16.7 | 22.3 | 5.6 | 16.7 |  |  |  |  |  |  |
| ^*^cleaners, helpers, construction  According to the International Standard Classification of Occupation  Percentages might not sum up to 100 % due to rounding. | | | | | | | |  |  |  |  |  |  |  |

| TABLE S2: Sleep timing and duration | | | | | | | | | | | | | |
| --- | --- | --- | --- | --- | --- | --- | --- | --- | --- | --- | --- | --- | --- |
| **Data from actimetry collected in the winter (March - September, n = 7 - 23)** | | | | |  |  |  |  |  |  |  |  |  |
|  | Quilombola communities | | | | | | |  | ANOVA results | | | |  |
|  | BB | AB | SR | CF | MM | MF | PB |  | F | df | p | n² |  |
| Sleep onset^*^ | 21:40 ± 0:38^a^ | 21:37 ± 1:04^a^ | 22:25 ± 1:06^a,b^ | 22:27 ± 1:50^a,b^ | 22:39 ± 1:03^a,b^ | 22:35 ± 0:59^a,b^ | 23:16 ± 0:57^b^ |  | 4.20 | 6, 76 | < 0.01 | 0.25 |  |
| Mid-sleep^**^ | 02:15 ± 0:31 | 02:17 ± 0:48 | 02:30 ± 0:45 | 02:54 ± 2:11 | 02:41 ± 0:56 | 03:06 ± 0:43 | 03:13 ± 0:44 |  | 2.36 | 6, 76 | < 0.05 | 0.16 |  |
| Sleep end^**^ | 06:49 ± 0:34 | 06:57 ± 0:35 | 06:34 ± 0:42 | 07:20 ± 2:37 | 06:43 ± 1:07 | 07:37 ± 0:41 | 07:12 ± 0:39 |  | 1.50 | 6, 76 | 0.19 | 0.11 |  |
| Sleep duration^*^ | 08:58 ± 0:38^a^ | 09:10 ± 0:38^a^ | 07:55 ± 0:58^a,b^ | 08:46 ± 1:06^a,b^ | 07:54 ± 1:06^b^ | 08:39 ± 1:00^a,b^ | 07:40 ± 0:55^b^ |  | 5.25 | 6, 76 | < 0.001 | 0.29 |  |
|  |  |  |  |  |  |  |  |  |  |  |  |  |  |
| **Data from the MCTQ on work-free days (n = 13 - 59)** | | | |  |  |  |  |  |  |  |  |  |  |
|  | Quilombola communities | | | | | | |  | ANOVA results | | | |  |
|  | BB | AB | SR | CF | MM | MF | PB |  | F | df | p | n² |  |
| Sleep onset^*^ | 21:42 ± 1:22^a^ | 21:57 ± 1:06^a,b^ | 23:24 ± 1:53^b,c^ | 22:27 ± 1:10^a,b^ | 22:48 ± 1:31^a,b,c^ | 23:46 ± 1:34^c^ | 23:54 ± 1:35^c^ |  | 10.09 | 6, 205 | < 0. 0001 | 0.22 |  |
| Mid-sleep^*^ | 02:06 ± 1:05^a^ | 02:31 ± 0:44^a,b^ | 03:01 ± 1:31^a,b,c^ | 02:46 ± 1:22^a,b,c^ | 02:36 ± 1:10^a^ | 03:40 ± 1:09^c^ | 03:36 ± 1:25^b,c^ |  | 7.60 | 6, 205 | < 0. 0001 | 0.18 |  |
| Sleep end^*^ | 06:30 ± 1:13^a^ | 07:06 ± 0:55^a,b^ | 06:39 ± 1:36^a,b^ | 07:05 ± 1:48^a,b^ | 06:24 ± 1:27^a^ | 07:40 ± 1:27^b^ | 07:18 ± 1:38^a,b^ |  | 3.77 | 6, 205 | < 0.01 | 0.10 |  |
| Sleep duration^*^ | 08:48 ± 1:25^a^ | 09:07 ± 1:24^a^ | 07:15 ± 1:45^b^ | 08:39 ± 1:16^a,b^ | 07:36 ± 1:51^a,b^ | 08:00 ± 1:34^a,b^ | 07:24 ± 1:31^b^ |  | 5.07 | 6, 205 | < 0. 0001 | 0.13 |  |
| Columns show the communities mean ± standard deviation.  * letters represent subsets with significant differences detected by Tukey post hoc.  ** differences detected by ANOVA, despite not having enough power for the post hoc comparisons to detect where.  *** for MCTQ comparisons: one outlier removed (group MF, ROUT method) | | | | | | | | | | | | |  |

| Table S3: Hierarchical regression - predictors of midpoint of sleep (N = 123) | | | | | | | | | | | | | | | | | | | |  |  |
| --- | --- | --- | --- | --- | --- | --- | --- | --- | --- | --- | --- | --- | --- | --- | --- | --- | --- | --- | --- | --- | --- |
|  |  | | **Model 1** | | | | | | **Model 2** | | | | | **Model 3** | | | | | |  |  |
|  |  | | Adjusted R² = -0.010 | | | | | | Adjusted R² = 0.190 | | | | | Adjusted R² = 0.302 | | | | | |  |  |
|  | | | F(2, 120) = 0.39, p = 0.68 | | | | | | F(8,114) = 4.58, p < 0.0001 | | | | | F(10,112) = 6.27, p < 0.0001 | | | | | |  |  |
| **Variables** | | | B | | β | p | | | B | | β | p | | B | | β | | p | |  |  |
| Age | | | 0.00 | | 0.04 | 0.66 | | | -0.01 | | -0.21 | < 0.05 | | -0.01 | | -0.27 | | < 0.01 | |  |  |
| Sex (male) | | | -0.13 | | -0.07 | 0.46 | | | -0.01 | | 0.00 | 0.96 | | 0.33 | | 0.17 | | 0.05 | |  |  |
| Quilombo (BB) | Areia Branca (AB) | |  | |  |  | | | 0.21 | | 0.07 | 0.49 | | 0.30 | | 0.10 | | 0.31 | |  |  |
|  | São Roque (SR) | |  | |  |  | | | 0.52 | | 0.20 | < 0.05 | | 0.13 | | 0.05 | | 0.61 | |  |  |
|  | Córrego do Franco (CF) | |  | |  |  | | | 0.76 | | 0.24 | < 0.05 | | 0.65 | | 0.20 | | < 0.05 | |  |  |
|  | Mamãs (MM) | |  | |  |  | | | 0.64 | | 0.25 | < 0.05 | | 0.51 | | 0.20 | | < 0.05 | |  |  |
|  | Morro do Fortunato (MF) | |  | |  |  | | | 1.36 | | 0.52 | < 0.0001 | | 1.51 | | 0.58 | | < 0.0001 | |  |  |
|  | Peixoto dos Botinhas (PB) | |  | |  |  | | | 1.34 | | 0.52 | < 0.0001 | | 1.17 | | 0.45 | | < 0.0001 | |  |  |
| Season of data collection (winter) | | |  | |  |  | | |  | |  |  | | -0.41 | | -0.21 | | < 0.05 | |  |  |
| Average light exposure during the day | | |  | |  |  | | |  | |  |  | | 0.00 | | -0.35 | | < 0.001 | |  |  |
| The model was carried out with midpoint of sleep derived from actimetry as dependent variable. Quilombo represents participant’s community. Season of data collection refers to when actimetry data were collected. Average light exposure during the day as measured by actimetry. | | | | | | | | | | | | | | | | | | | |  |  |
|  |  | |  | |  |  | | |  | |  |  | |  | |  | |  | |  |  |
| Table S4: Hierarchical regression - predictors of sleep duration (N = 123) | | | | | | | | | | | | | | | | | | | |  |  |
|  |  | | **Model 1** | | | | | | **Model 2** | | | | | **Model 3** | | | | | |  |  |
|  |  | | Adjusted R² = 0.018 | | | | | | Adjusted R² = 0.218 | | | | | Adjusted R² = 0.307 | | | | | |  |  |
|  | | | F(2, 120) = 2.13 p = 0.12 | | | | | | F(8,114) = 5.24, p < 0.0001 | | | | | F(10,112) = 6.41, p < 0.0001 | | | | | |  |  |
| **Variables** | | | B | | β | p | | | B | | β | p | | B | | β | | p | |  |  |
| Age | | | -0.01 | | -0.18 | <0.05 | | | -0.01 | | -0.12 | 0.22 | | -0.01 | | -0.10 | | 0.30 | |  |  |
| Sex (male) | | | -0.10 | | -0.05 | 0.56 | | | -0.17 | | -0.09 | 0.29 | | -0.41 | | -0.21 | | < 0.05 | |  |  |
| Quilombo (BB) | Areia Branca (AB) | |  | |  |  | | | 0.28 | | 0.08 | 0.40 | | 0.23 | | 0.07 | | 0.46 | |  |  |
|  | São Roque (SR) | |  | |  |  | | | -0.80 | | -0.29 | < 0.01 | | -0.41 | | -0.15 | | 0.14 | |  |  |
|  | Córrego do Franco (CF) | |  | |  |  | | | -0.40 | | -0.12 | 0.20 | | -0.25 | | -0.07 | | 0.41 | |  |  |
|  | Mamãs (MM) | |  | |  |  | | | -1.12 | | -0.41 | < 0.0001 | | -0.91 | | -0.33 | | < 0.001 | |  |  |
|  | Morro do Fortunato (MF) | |  | |  |  | | | -0.29 | | -0.10 | 0.36 | | -0.36 | | -0.13 | | 0.23 | |  |  |
|  | Peixoto dos Botinhas (PB) | |  | |  |  | | | -1.01 | | -0.36 | < 0.01 | | -0.81 | | -0.29 | | < 0.01 | |  |  |
| Season of data collection (winter) | | |  | |  |  | | |  | |  |  | | 0.58 | | 0.29 | | < 0.001 | |  |  |
| Average light exposure during the day | | |  | |  |  | | |  | |  |  | | 0.00 | | 0.21 | | < 0.05 | |  |  |
| The model was carried out with sleep duration derived from actimetry as dependent variable. Quilombo represents participant’s community. Season of data collection refers to when actimetry data were collected. Average light exposure during the day as measured by actimetry.  TABLE S5: Photoperiod and climate | | | | | | | | | | | | | | | | | | | | | |
|  | | | Winter solstice | | Summer solstice^*^ | | | | Closest meteorological station | | | | | Monthly insolation (h) | | Relative  humidity (%) | | Nebulosity^**^ | |  |  |
| Bombas (BB) | | | 06:23 - 17:11 | | 05:10 - 18:24 | | | | Curitiba (PR) | | | | | 151.80 | | 80.41 | | 7.21 | |  |  |
| Areia Branca (AB) | | | 06:25 - 17:09 | | 05:08 - 18:26 | | | | Curitiba (PR) | | | | | 151.80 | | 80.41 | | 7.21 | |  |  |
| São Roque (SR) | | | 06:29 - 16:57 | | 04:56 - 18:30 | | | | Torres (SC) | | | | | 176.33 | | 83.91 | | 6.12 | |  |  |
| Córrego do Franco (CF) | | | 06:25 - 17:09 | | 05:08 - 18:26 | | | | Curitiba (PR) | | | | | 151.80 | | 80.41 | | 7.21 | |  |  |
| Mamãs (MM) | | | 06:21 - 17:05 | | 05:04 - 18:22 | | | | Castro (PR) | | | | | 117.03 | | 83.59 | | 7.04 | |  |  |
| Morro do Fortunato (MF) | | | 06:31 - 17:03 | | 05:02 - 18:32 | | | | Florianópolis (SC) | | | | | 168.46 | | 79.04 | | 6.71 | |  |  |
| Peixoto dos Botinhas (PB) | | | 06:23 - 16:47 | | 04:47 - 18:24 | | | | Porto Alegre (RS) | | | | | 178.30 | | 77.57 | | 5.64 | |  |  |
| Photoperiod as calculated using ChronoSapiens  Meteorological data available from Instituto Nacional de Metereologia (INMET). Monthly averages from 2013-2016. | | | | | | | | | | | | | | | | | | | | | |
| ^*^no Daylight Saving Time advance  ^**^Fraction of sky covered by clouds (1-10) | | | | | | | |  | | |  | | |  | |  | |  | | | |


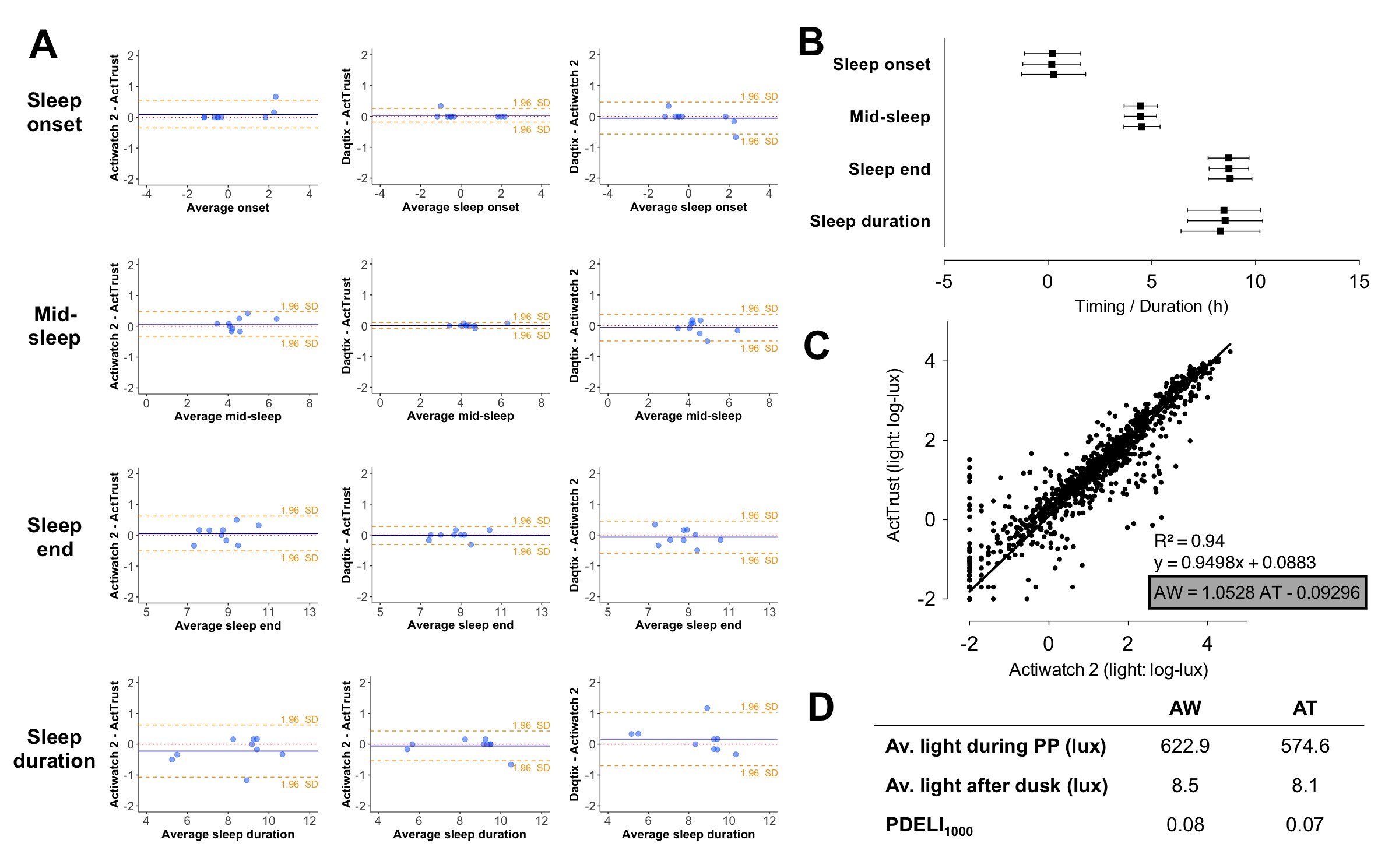


***Figure S1. Comparison between Actiwatch 2 (Philips Respironics) and ActTrust (Condor).*** *(a) Bland-Altman plots for comparing Actiwatch 2 and ActTrust, Daqtix and ActTrust, and Daqtix and Actiwatch. The blue line represents the mean difference between all days (n = 9) recorded with the two different devices worn at the same time on the same wrist. The orange dashed lines represent 1.96 standard deviation. (b) Squares and whiskers represent the calculated average and standard deviation of recorded days for each variable (sleep onset, mid-sleep, sleep end and sleep duration) and each actimeter. Top: Actiwatch 2; Middle: Daqtix; Bottom: ActTrust. (c) The grey square shows the slope equation from* *data collected over 14 days using Actiwatch 2 and ActTrust: 1657 bins of 10 min, representing 276 hours of recordings shown on the scatterplot. This was used to normalise daily averages derived from ActTrust. (D) Daily averages of light exposure during photoperiod (PP), daily averages of light after dusk and PDELI_1000_ calculated from data from both actimeters were not significantly different (Wilcoxon Matched-pairs signed rank test, average light during photoperiod: Z = - 22, p = 0.37; light after dusk: Z = 12, p =0.64).*


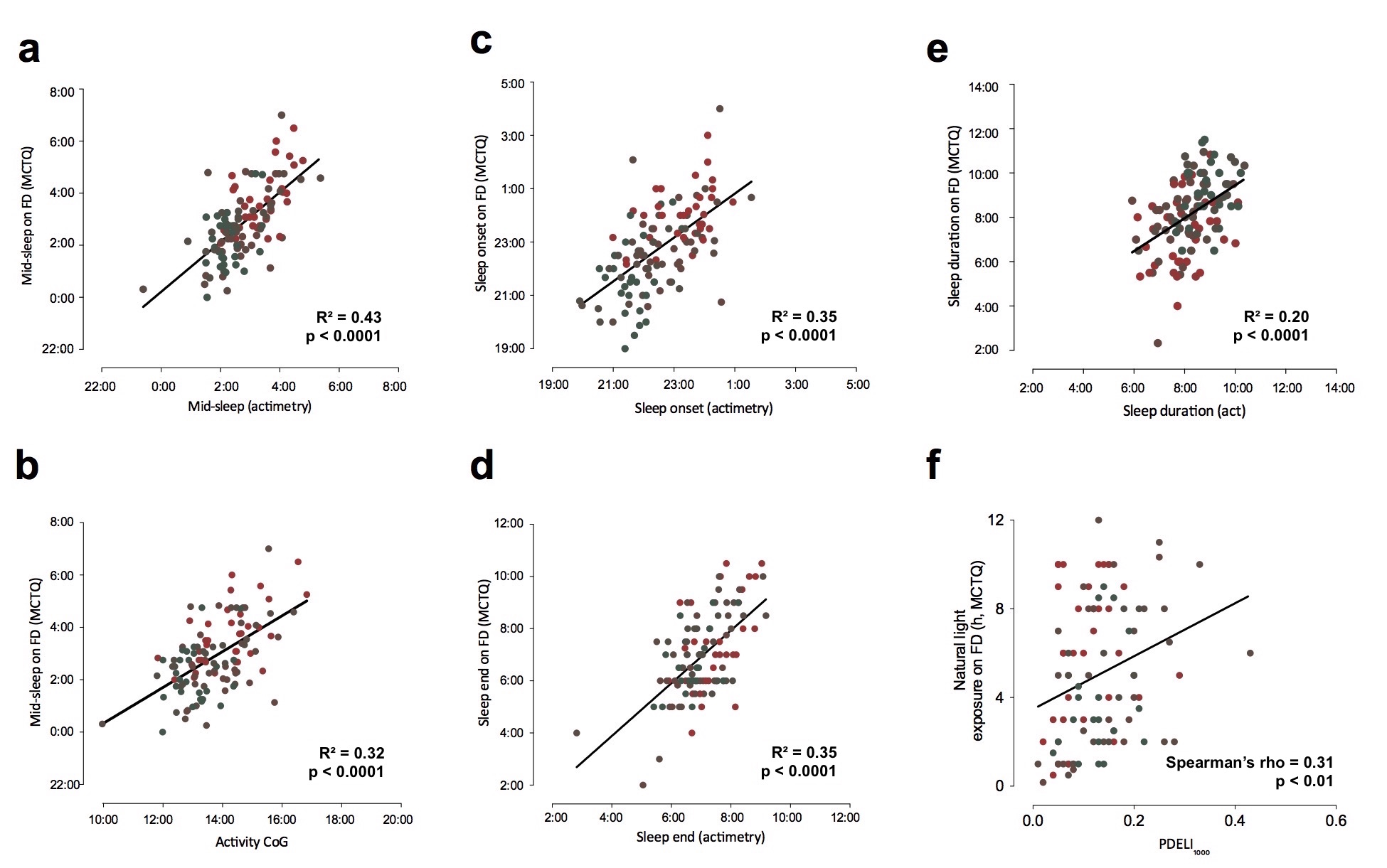


*Figure S2. Correlation between subjective data from the MCTQ and actimetry. MCTQ-derived mid-sleep on free days correlated significantly with mid-sleep calculated from actigraphy data and the centre of gravity of activity (Pearson: a, mid-sleep: r = 0.66, p < 0.0001; b, activity CoG: Pearson, r = 0.57, p < 0.0001, N = 117). The same was true for sleep onset, end and duration (Pearson, c, onset: r = 0.60, p < 0.0001; d, end: r = 0.59, p < 0.0001; e, duration: r = 0.45, p < 0.0001, N = 117). MCTQ assessed natural light exposure correlated with the proportion of day exposed to more than 1000 lux (PDELI_1000_, Spearman, rho = 0.31, p < 0.01, N = 99). Dots are colour coded according to the geographical isolation index. The greener the dots, the more geographically isolated the community.*
